# Children and Young People Presenting in a Pediatric Emergency Department in North-West England in Suicidal Crisis: An Exploratory Case Series Study

**DOI:** 10.3389/fpsyt.2022.892939

**Published:** 2022-04-25

**Authors:** Emma Ashworth, Serena Provazza, Molly McCarthy, Pooja Saini

**Affiliations:** Faculty of Health, School of Psychology, Liverpool John Moores University, Liverpool, United Kingdom

**Keywords:** child and adolescent, suicide, crisis, self-harm, emergency departments, mental health

## Abstract

Suicide is a leading cause of death among children and young people (CYP) worldwide, and rates have been increasing in recent years. However, while evidence exists regarding factors associated with suicide and self-harm, there is limited information publicly available on the CYP who present in suicidal crisis. This is a case series study of CYP (aged 8–16) experiencing suicidal crisis who presented in an Emergency Department at a pediatric hospital in North-West England between March 2019 and March 2021 (*n* = 240). Clinical records were extracted and audited to explore demographic data, methods of recording patient attendance, the clinical pathways available and the patterns of pathway usage, and differences in CYP presentations before and after the COVID-19 pandemic. Attendees were mostly White females, with a mean age of 13.5 years, and 24% had a diagnosed special educational need. “Social/social problems” was the most commonly used code for recording attendance (38%), and pathways varied depending on code used. A range of parental and familial factors were also identified. There were more CYP presenting with self-harm in addition to suicide ideation after the pandemic began (43 vs 27% pre-pandemic). This study provides the first clear insight into CYP who seek help at a North-West Emergency Department for suicidal crisis, and work is now needed to develop effective prevention strategies tailored toward the groups most at-risk.

## Introduction

Suicide is still the leading cause of death among children and young people (CYP) in the United Kingdom (1–3), with suicide rates amongst adolescents having increased by 7.9% per year in the last decade (4). Rates have increased even further in more recent years; 2018 data on suicide deaths from the Office for National Statistics (ONS) indicate a 22% one-year increase in suicide rates in under 25-year-olds, the largest rise amongst all age groups (5). In adolescents and young adults, rates of death by suicide are 2–4 times higher in men than in women, while suicide attempts are 3–9 times more common in women than men (6, 7). Within the context of suicide research, these gender differences in suicidal behavior rates are known as the “Gender Paradox” (8). In adolescents and young adults, this paradox changes according to age (9); women’s suicide attempt rates increase with age, peaking in mid-adolescence, whereas men’s suicide rates continue to increase into early adulthood (10, 11). The United Kingdom suicide rate in adolescent girls is now the highest since records began in 1981 (12, 13). Furthermore, there is some emerging evidence of a possible trend of increasing child suicide deaths in England during the COVID-19 pandemic and associated lockdowns, although this is provisional and numbers are too small for any meaningful analysis (14).

Several risk factors may increase the likelihood of suicide in CYP; indeed suicide is thought to be caused by the interplay of genetic, biological, psychological, and social factors (15). Research has identified various risk factors associated with youth suicide (15, 16) including previous or recent stresses such as witnessing domestic violence, bullying, self-harm, bereavement (including by suicide), academic pressures (13), and special educational needs (SEN), in particular Autism Spectrum Condition (ASC). Population-based mortality studies have demonstrated extraordinarily high rates of death by suicide in autistic youth and adults (17). Gender differences have been identified in this population as well, with higher rates of suicide attempts for autistic girls compared with boys (17).

Research suggests that outcomes for autistic girls are worsened by poor understanding of the differences in autism presentation between genders, including greater levels of camouflaging or masking behavior to conceal autistic characteristics, and better social communication and interaction among autistic girls (18). This gender bias is further exasperated by ill-informed diagnostic criteria and the development and validation of assessment tools that fail to tap into the “female phenotype,” due to most materials being validated with males. Subsequently, vital opportunities to diagnose autistic girls are missed, leading to under- and late diagnosis, particularly for those without intellectual disability. Thus, girls are diagnosed with autism at later ages than boys, with many women getting their first diagnosis well into adulthood (19–21). The delayed diagnosis and subsequent support offered to autistic girls may explain the high rate of suicide attempts in this population (17).

Aside from socio-demographic characteristics, another well-established, yet scarcely investigated, psychological risk factor for death by suicide is suicide ideation or crisis (16). Suicidal crisis is a spectrum, ranging from thoughts of death and passive ideation with no intent or plan, to specific suicidal ideation with intent or plan (16). It has been shown that the more pervasive the suicidal crisis, the more likely the individual is to attempt suicide (16, 22). Evidence suggests that around 80% of individuals who have died by suicide did seek help for crisis at least once in the year before their death, and most of them had Emergency Department (ED) contact (23).

Risk factors or correlates associated with suicidal crisis are given substantially less attention in the extant evidence base than factors associated with suicide attempts or completed suicides. Although a meta-analysis by Ribeiro et al. (24) did identify risk factors in five subcategories for suicide ideation (prior suicide ideation, hopelessness, depression diagnosis, abuse history, and anxiety diagnosis), they found that prediction was only slightly better than chance for all outcomes, and no broad (sub)category accurately predicted far above chance levels; this has not changed in the past 50 years. There is also minimal evidence in a United Kingdom setting, particularly with hospital-based samples or with CYP specifically. Furthermore, the studies that have been conducted with CYP often look at individualistic trait risk factors, such as perceived burdensomeness, hopelessness, and stress (25), even though these factors studied in isolation are usually not useful in predicting suicide risk (26). Research has generally failed to explore pervasive socio-demographic risk factors for suicidal crisis or ideation in broader ecological domains.

However, evidence suggests that risk factors for or correlates of adolescent suicide *attempts* do operate within multiple socio-ecological domains in a young person’s environment (26, 27). In keeping with Bronfenbrenner’s Ecological Systems Theory [EST; (28)], evidence exists for risk factors relating to abuse, parental substance misuse, bullying, and chronic familial dysfunction or violence [see (29) for an overview]. According to EST, risk factors in these socio-ecological domains interact with and impact one another in a complex system (30), influencing an individual either directly or indirectly. Thus, family and community factors are also integral (31). Ayyash-Abdo (26) attempted to apply EST to adolescent suicides, identifying key risk factors in each system. In the microsystems (i.e., proximal risk factors), factors in the familial (e.g., family history of suicides, parental psychopathology, loss), peers (e.g., loneliness, low levels of peer support) and school (e.g., academic performance, perceived school connectiveness) domains were also identified, in addition to the individual factors such as depression, hopelessness, and drug and alcohol use. Furthermore, in the macrosystems (i.e., distal risk factors), factors such as the media, and ethnic, cultural, and societal differences all influenced adolescents’ suicide risk. However, while there is evidence to suggest that there is some overlap between risk factors for suicide and suicidal crisis (32), there is also some evidence indicating distinctions between them (29). Thus, further work is needed in this area.

Despite evidence that suicidal crisis is a risk factor contributing to suicide among CYP, the number of presentations for suicidal crisis without attempts at EDs is not consistently registered, nor is there consistent coding used across NHS Trusts for recording patients who present at ED in suicidal crisis (33). Therefore, while national data is already available for individuals who attend ED for self-harm (34), there is a lack of national data available for those individuals who attend ED in suicidal crisis. Given the relationship between suicidal crisis and later suicide attempts, a consistent code for suicidal crisis, and an understanding of the factors that are associated with suicidal crisis, are of crucial importance in the prevention of future deaths. This information would provide services with a better understanding of the number of CYP in suicidal crisis, which in turn could lead to a more effective management of such individuals, as well as reduced youth suicide rates (33, 35).

In addition, there is also a lack of consistent evidence regarding the pathways available across NHS Trusts for individuals who present to ED in suicidal crisis (36). Clinical pathways available for CYP who attend in suicidal crisis tend to be complex and they have not previously been examined systematically, despite evidence which suggests that prompt referrals to clinical pathways and application of appropriate interventions can empower hospital systems in the management and prevention of suicide (1, 37). Thus, a rigorous evaluation of the pathways available for CYP who attend EDs in suicidal crisis is needed, to inform better modeling of service provision for these patients (37).

## The Current Study

The North-West of England has a suicide rate that falls around the national average, with 10.7 deaths per 100,000 (national average = 10.4) although this varies considerably across different areas of the region (12). Suicide rates among CYP are not reported by area, and so self-harm is the closest proxy indicator, given that 52% of CYP who die by suicide have previously self-harmed (38). Hospital admissions rates for self-harm for 10–24 year olds are significantly worse in the North-West region compared to the England average (520.5 vs 430.5 per 100,000), and rates are particularly concerning for 15–19 year olds (39).

One dedicated pediatric hospital in the region has its own children’s ED and Child and Adolescent Mental Health Services (CAMHS), including community CAMHS, in- and out-patient clinics, and a dedicated CAMHS crisis team. The crisis service includes a multi-disciplinary team who provide support to CYP presenting in crisis regarding self-harm, suicidal ideation, and acute mental health difficulties. However, until now, no formal analysis of the hospital’s data has been conducted into the number of CYP presenting at ED in suicidal crisis, the demographic characteristics of those presenting, the subsequent pathways that they follow, or how ED presentations are recorded in the hospital’s system. Furthermore, while anecdotal evidence indicates a sharp increase in demand on the crisis team’s services since the COVID-19 pandemic began in March 2020, significant differences in ED attendance before and after the pandemic have not been explored.

To address this, we aimed to compile data pertaining to the number of CYP presenting at the hospital’s ED in suicidal crisis, how this was coded, and the resultant care pathways they followed. An audit was then conducted of the hospital’s ED data for all CYP who had presented in suicidal crisis in the years 2019–2021. The study aimed to address the following research questions:

1. Are certain socio-demographic characteristics across ecological domains significantly associated with ED attendance for suicidal crisis among CYP?
2. What are the most common methods for recording presentations of suicidal crisis in this ED?
3. What are the clinical pathways available to CYP who attend the ED in suicidal crisis, and what are the patterns of pathway usage?
4. Are there differences in the characteristics of CYP who attend the ED in suicidal crisis before and after the start of the COVID-19 pandemic in March 2020?

## Materials and Methods

### Design and Setting

This retrospective case series study included CYP experiencing suicidal crisis who had attended an ED at a local pediatric hospital between March 2019 and March 2021 (*n* = 240). Access to the anonymised data was approved by the hospital’s research department.

### Participants and Data Extraction

Clinical records at the hospital were reviewed between March 2019 and March 2021. Inclusion criteria included any patients aged 16 or younger (the hospital advises anyone age over 16 to attend an adult ED) who presented to ED in suicide crisis (with and without self-harm) during the study period. Data on CYP that visited the hospital in suicide crisis were provided to the researcher by the hospital data team. An electronic inspection of the clinical notes was performed through the Meditech system (Medical Information Technology Inc., Westwood, MA, United States). All patient notes under potentially relevant codes (e.g., low mood, suicide thoughts, social problems, overdose) were audited, and those indicating suicidal crisis or ideation were extracted, collated, and anonymised. Each patient’s clinical record was inspected and included in the study only if suicide crisis or ideation was clearly reported in the clinical notes.

Variables examined included sex, ethnicity, SEN, presence of suspected ASC traits, mental health conditions, suicide ideation with or without self-harm, history of self-harm, clinician determined risk (in terms of Pierce score), and parental socio-demographics. It was also investigated whether the children were previously known to CAMHS or were under CAMHS at the time of the ED presentation. These data were either collected from the family using a standard *pro forma* completed by the clinician when triaging the patient, or they were already available on the hospital system if the patient had previous been under Community Pediatrics and/or was currently known to CAMHS. Further details regarding the demographics of the patients are presented in the results section below.

### Data Analysis

Our sample size was predetermined based on the number of CYP attending ED. This was an exploratory analysis, whereby descriptive statistics were conducted to identify the socio-demographics of the sample and the factors characteristic of CYP presenting in suicidal crisis. Chi-squared analyses, regressions, and independent samples *t*-tests were also conducted to establish statistically significant associations and differences in the dataset. We chose not to conduct Bonferroni corrections for multiple comparisons based on recommendations from Armstrong (40) and Rothman (41) that corrections for multiple comparisons in exploratory studies are not required, due to the increased likelihood of Type 2 errors. Analyses were conducted in IBM SPSS 26.

While the researchers had access to all records, the dataset only captured entries made in clinical records; unrecorded clinical activity or missing information from ED documents was therefore unavailable. For the purposes of this study, only the presence of each factor within each person’s clinical records was used for the analysis. It is possible this strategy may have led to underestimation of some factors: for example, sexual orientation.

## Results

### Socio-Demographic Characteristics of Patients: Individual Factors

#### Demographic Characteristics

Between March 2019 and 2021, 240 CYP attended the hospital’s ED for suicidal crisis (see Table 1). The majority of attendees were female (67%; *n* = 160) and White British (93%; *n* = 222), and the mean age was 13.5 years (SD = 1.42; range = 8–16).

**TABLE 1:** Individual socio-demographic characteristics of patients presenting in suicidal crisis.

Demographic	*N*	Percentage of whole sample
**Sex**		
Female	160	66.6
Male	80	33.3
**Ethnicity**		
White British	222	92.5
Other	16	6.7
Unknown	2	0.8
**SEN**		
Yes	58	24.2
ADHD	12	5.0
ADHD and learning disabilities	3	1.3
ASC	21	8.8
ASC and ADHD	12	5.0
ASC, ADHD and learning disabilities	1	0.4
ASC and learning disabilities	3	1.3
Learning disabilities	6	2.5
No	182	75.8
**ASC Traits**		
Yes	51	21.3
No	189	78.8
**Previous mental health difficulties**		
Yes	142	59.2
Anxiety	43	17.9
Anxiety and comorbidities	10	4.2
Anxiety and low mood	9	3.8
Low mood	40	16.7
Low mood and comorbidities	5	2.1
Other	35	14.6
No	97	40.4
Unknown	1	0.4
**Previously known to CAMHS**		
Yes	154	64.2
No	86	64.2
**Currently under CAMHS**		
Yes	54	22.5
No	186	77.5
**History of self-harm**		
Yes	162	67.5
No	78	32.5
**Clinician-determined risk**		
Low	37	15.4
Moderate	44	18.3
High	51	21.3
n/a	26	10.8
Unknown	82	34.2

Approximately one-quarter of CYP had a diagnosed SEN (24%; *n* = 58). 10% (*n* = 6) of the patients with an SEN had a diagnosis of learning disabilities, 41% (*n* = 24) had ASC with/without other learning disabilities, 26% (*n* = 15) had attention deficit hyperactivity disorder (ADHD) with/without other learning disabilities, and 22% (*n* = 13) had both ASC and ADHD.

However, an additional 51 CYP (21%) from the whole sample (i.e., those with and without a diagnosed SEN, but excluding those with an existing ASC diagnosis) were recorded as having suspected ASC traits (as suspected by the clinician assessing the patient or based on the patient currently being on the ASC pathway awaiting diagnosis), meaning that in total 37% (*n* = 88) of the whole sample had either ASC or ASC traits. Further exploration of this data indicated that 61% (*n* = 54) of those with ASC or ASC traits were female.

#### Mental Health History

The majority of CYP had a history of mental health difficulties (60%; *n* = 142). Of those with existing difficulties, the most common diagnoses were anxiety with/without comorbidities (44%; *n* = 62) or low mood with/without comorbidities (32%; *n* = 45), although the majority had no formal diagnosis recorded (69%; *n* = 98). Most patients were previously known to CAMHS (64%; *n* = 154), and 23% (*n* = 54) were currently under CAMHS when they attended the ED (see Table 1).

The majority of CYP did not have a previous ED attendance for suicidal crisis (76%; *n* = 183). However, 69% (*n* = 162) had a history of self-harm. Clinician-determined risk (based on the Pierce Suicide Intent Scale, a standardized measure of risk of death by suicide) was deemed high for 21% of the sample (*n* = 51), moderate for 18% (*n* = 44) and low for 15% (*n* = 37). Risk data, however, should be interpreted with caution due to the large proportion (34%; *n* = 82) of “unknown” recordings, and the limited data available on the reliability of the measure for use with CYP. Risk levels were significantly associated with sub-diagnosis [X(4) = 51.65, *p* < 0.001 φ = *0.46*], whereby higher risk levels were given to patients who had also self-harmed.

### Socio-Demographic Characteristics of Patients: Familial Factors

The majority of CYP presenting with suicidal crisis reported separation or loss of a parent (68%; *n* = 162), and most were living with a single parent or a single parent and siblings (44%; *n* = 106). Parental mental health issues (44%; *n* = 105), parental drug misuse (17%; *n* = 41), and parental criminality (16%; *n* = 38) were also recorded among a sizeable minority of CYP, although a large proportion of data were missing for these variables. Approximately one-quarter of CYP had experienced neglect (24%; *n* = 57) or domestic violence (24%; *n* = 57), and one-third experienced some form of physical, emotional and/or sexual abuse (32%; *n* = 77). 23% were known to a social worker (see Table 2).

**TABLE 2:** Familial socio-demographic characteristics of patients presenting in suicidal crisis.

Demographic	*N*	Percentage of whole sample
**Separation or loss of a parent**		
Yes	164	68.3
No	70	29.2
Unknown	6	2.5
**Living Circumstances**		
Both parents (with/without siblings)	67	27.9
Parent and step-parent (with/without siblings)	24	10.1
Single parent (with/without siblings)	106	44.2
Other	32	13.3
Care home	1	0.4
Unknown	10	4.2
**Parental mental health difficulties**		
Yes	105	43.8
No	93	17.5
Unknown	42	17.5
**Parental drug misuse**		
Yes	41	17.1
No	150	62.5
Unknown	49	20.4
**Parental criminality**		
Yes	38	15.8
No	154	64.2
Unknown	48	20.0
**Neglect**		
Yes	57	23.8
No	136	56.7
Unknown	47	19.6
**Domestic violence**		
Yes	57	23.8
No	135	56.3
Unknown	48	20.0
**Abuse**		
Yes	77	32.1
No	117	48.8
Unknown	46	19.2
**Social worker**		
Yes	55	22.9
No	164	68.3
Unknown	21	8.8

Further examination of parental factors revealed a number of significant associations with CYP’s mental health diagnosis (if they had received one), as displayed in Table 3. A statistically significant association was found between parental mental health (reported by parents) and CYP’s previous mental health diagnoses [*X*(4) = *15.30, p* = *0.004*]; as such a young person was significantly more likely to have been diagnosed with mental health difficulties if their parent also had mental health difficulties. A significant association was also found between parental drug misuse [*X*(4) = *13.92, p* = *0.008*], parental criminality [*X*(4) = *13.62, p* = *0.009*] and CYP mental health diagnosis. Results suggested those CYP whose parents had misused drugs or reported criminality were more likely to have diagnosed mental health difficulties. Furthermore, those CYP with diagnosed mental health difficulties were also statistically significantly more likely to have experienced neglect [*X*(4) = *16.87, p* = *0.002*], have witnessed domestic violence [*X*(4) = *12.68, p* = *0.013*], abuse [*X*(4) = *14.84, p* = *0.005*], and have experienced parental separation or loss [*X*(4) = *12.23, p* = *0.016*].

**TABLE 3:** Chi-square analysis of the association between CYP diagnosed mental health difficulties and parental risk factors.

Variable	X	df	*p*	Cramer’s V
Parental Mental Health	15.30	4	0.004**	0.179
Parental Drug Misuse	13.92	2	0.008**	0.170
Parental Criminality	13.62	4	0.009**	0.168
Neglect	16.87	4	0.002**	0.187
Domestic Violence	12.68	4	0.013*	0.163
Abuse	14.84	4	0.005**	0.176
Parental Separation or Loss of a Parent	12.23	4	0.016*	0.160

*p < 0.05; **p < 0.01.

### Methods of Recording ED Presentations by Clinicians

#### Diagnosis at ED

In total, 65% of patients received a diagnosis of “suicide ideation” (*n* = 157) when presenting at the ED. The remainder received a diagnosis of “suicide ideation with deliberate self-harm” (35%; *n* = 83). Of those who presented with ideation and deliberate self-harm, the most common means were overdose (55%; *n* = 46) cutting (28%; *n* = 23), and suffocation (10%; *n* = 8).

#### Coding

“Social problems” was the most commonly used code to record attendances at the ED for suicidal crisis (see Table 4), with 22% of all attendances recorded under that code (*n* = 53). This was followed by the codes “other” (21%; *n* = 51), “social” (15%; *n* = 53), and “overdose” (13%; *n* = 32).

**TABLE 4:** Diagnosis and coding frequencies for patients with suicidal crisis.

Recording	*N*	Percentage of whole sample
**Diagnosis**		
Suicide ideation	157	65.4
Suicide ideation with deliberate self-harm	83	34.6
Cutting	23	9.6
Overdose	46	19.2
Suffocation	8	3.3
Other	6	2.5
**Code**		
Low mood	3	1.3
Mental health	19	7.9
Other	51	21.3
Overdose	32	13.3
Self-harm	21	8.8
Social	37	15.4
Social problem	53	22.1
Suicidal thoughts	24	10.0

A regression analysis was conducted with “code” as the dependent variable, and socio-demographic characteristics as the independent variables. SEN diagnosis status (β = 0.163, *p* = 0.021), social worker status (β = −0.148, *p* = 0.034), and sub-diagnosis (β = 0.24, *p* = 0.040) were significant predictors of coding type. Further analysis of means indicated that individuals diagnosed with an SEN were more likely to be given the code of “other” compared to those without an SEN [*X*(1) = *6.05, p* = *0.014, φ* = *0.16*]. Having a social worker was associated with less use of the “overdose” category [*X*(2) = *9.52, p* = *0.009, φ* = *0.20*]. Having a sub-diagnosis of “suicide ideation with deliberate self-harm” (compared to “suicide ideation”) was associated with increased use of the “overdose” category [*X*(1) = *51.26, p* < *0.001, φ* = *0.46*], “deliberate self-harm category” [*X*(1) = *21.87, p* < *0.001*, φ = *0.30]*, and less use of the “suicidal thoughts” category [*X*(1) = *8.12, p* = *0.004, φ* = *0.35]* and “social” and “social problems” categories [*X*(1) = *28.74, p* < *0.001, φ* = *0.35*].

### Patterns of Clinical Pathway Usage

#### Initial Referral

After presenting at the ED, the majority of young people were admitted to an inpatient ward (45%; *n* = 107) or were seen as an outpatient (30%; *n* = 73). From December 2020 the Crisis Care Team increased liaison with the ED, meaning that CYP were assessed by a member of the Crisis Care team (once deemed fit), to evaluate current risk and mental state. Based on the outcome of the assessment, CYP were referred to the most appropriate service or discharged back to the community. A full list of referrals are presented in Table 5.

**TABLE 5:** Referral pathways for patients in ED with suicidal crisis.

Initial Referral	*N*	Proportion of whole sample
Admitted inpatient ward	107	44.6
ED assessment by Crisis Care Team	28	11.7
Discharged	29	12.1
Left before seen	2	0.8
Outpatient	73	30.4
n/a	1	0.4

**Referral Pathway**	***N***	**Proportion of whole sample**

Admitted to another service	2	0.8
Already on CAMHS waiting list	7	2.9
Already under another service/no further treatment appropriate	5	2.1
Discharged/signposted to another service	15	6.3
Follow-up	46	19.2
Follow-up by local CAMHS	49	20.4
Referred to another service/specialty	35	14.6
Referred to local CAMHS	78	32.5
n/a	3	1.3

#### Referral Pathway

Following the young person’s attendance to the ED, eight referral pathways were utilized. The most frequently used was “referral to local CAMHS” (33%; *n* = 78), followed by “followed up by local CAMHS” (20%; *n* = 49) and “follow-up” (19%; *n* = 46). A full list of referral pathways is presented in Table 5.

A chi-squared test was used to further analyze the referral pathways for young people attending the ED in suicidal crisis. A significant association was identified between the type of code recorded and the referral pathway the young person followed *[X*(56) = *88.46, p* = *0.004, φ* = *0.23]*, meaning the code assigned to the ED presentation significantly influenced where the patient was referred onto. For example, individuals coded as “social problems” were more likely to be referred to local CAMHS than to have no further treatment. However, no significant association was identified between gender [*X*(8) = *14.85, p* = *0.060, φ* = *0.249*] or age [*X*(64) = *63.19, p* = *0.505, φ* = *0.18]* and the referral pathway.

#### Outcomes for CYP With ASC Traits

Given the unexpectedly large proportion of individuals with ASC or ASC traits in the sample, exploratory chi-square tests were conducted to further examine the outcomes for this group of CYP. There was a significant association between ASC traits and referral pathway [*X*(8) = *16.59, p* = *0.035, φ* = *0.26*], suggesting that those with ASC traits were more likely to be followed-up by local CAMHS, whereas those with no ASC traits were more likely to be referred to local CAMHS.

**FIGURE 1:**
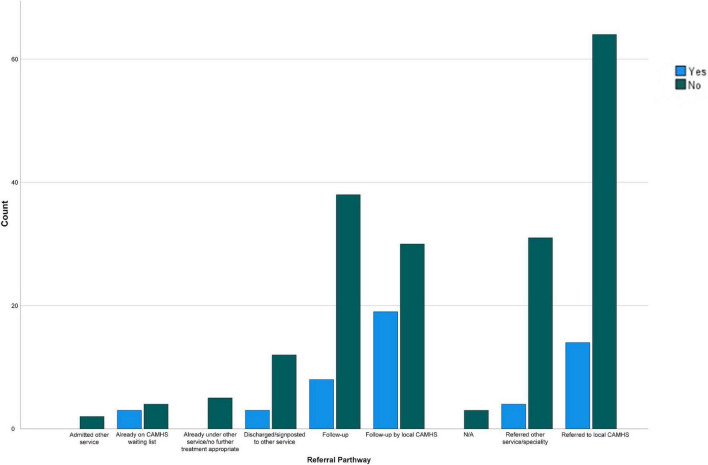
Referral pathway following ED attendance for suicidal crisis and ASC traits.

A significant association was also identified between an individual having ASC traits and them being already under CAMHS [*X*(1) = *9.32, p* = *0.002, φ* = *0.20*]; as such those presenting to the ED with ASC traits were more likely to already be known to CAMHS than those presenting with no ASC traits. All other tests were not significant (see Figure 1).

### Pre and Post COVID-19 Pandemic

Based on descriptive statistics, a number of differences were identified between March 2019–2020 and March 2020–2021. As the first COVID-19 lockdown occurred in March 2020 in the United Kingdom, this allows for a comparison pre and post the COVID-19 pandemic beginning. The mean age of the sample was broadly similar between the 2 years (13.48 vs 13.56). The number of females attending slightly increased from 64% in 2019–2020 to 69% in 2020/2021, whereas the number of males slightly reduced (36 to 31%). More CYP were previously known to CAMHS in the year following the pandemic beginning (69 vs 60%), and more were currently under CAMHS (29 vs 17%).

A series of independent *t*-tests were conducted to establish if there were any significant differences in attendees’ characteristics before and after the pandemic beginning. There were significant differences in the number of patients who had a social worker [*t*(227) = *−2.0, p* = *0.048*], and were currently under CAMHS [*t*(219) = *−0.2.27, p* = *0.024*], whereby more CYP were known to a social worker (28.1 vs 18.3%) and were under CAMHS (28.9 vs 16.7%) in the year after the pandemic began. There was also a significant difference in sub-diagnosis [*t*(228) = *−2.61, p* = *0.010*], with significantly more CYP presenting with suicide ideation with deliberate self-harm after the pandemic (43%) than before (27%).

In terms of referral pathways, there were significant differences between the 2 years regarding the number of CYP who were followed-up by CAMHS [*t*(218) = *−2.15, p* = *0.033*], and who were referred to other services [*t*(219) = *2.89, p* = *0.004*]. Specifically, a higher number of CYP were followed-up by CAMHS after the pandemic (26%) compared to the year before (15%), whereas a higher number of CYP were referred to other services or specialties in the year before the pandemic (21%) than afterward (8%). No other differences were statistically significant.

**FIGURE 2:**
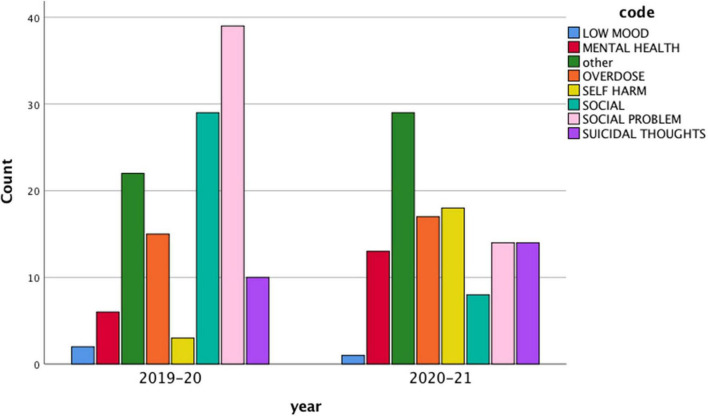
Code assigned to ED attendance for suicidal crisis by year: 2019–2020 and 2020–2021.

Chi-square analyses also indicated a significant association between year of attendance and code used [*X*(7) = *38.59, p* < *0.001, φ* = *0.40*], with the code “social problems” being used more in the year before the pandemic (31%) than afterward (12%). This is illustrated in Figure 2.

## Discussion

This case series study of a pediatric hospital’s ED in North-West England aimed to explore the socio-demographic characteristics of CYP attending for suicidal crisis, methods of recording presentations, the clinical pathways used, and differences in CYP presenting in the ED since the COVID-19 pandemic. Analyses indicated that 240 CYP attended the ED between March 2019 and March 2021. Attendees were mostly White females, with a mean age of 13.5 years. One-quarter had a diagnosed SEN, and almost one-third had either diagnosed or suspected ASC. Most had a history of mental health difficulties, most commonly anxiety, as well as deliberate self-harm, and were previously known to CAMHS. The majority also reported separation or loss of a parent; parental mental health issues, drug misuse, and criminality were also common. Additionally, abuse was frequently reported, including neglect or physical, emotional, and/or sexual abuse, as well as experiences of domestic violence. Almost one-quarter were known to a social worker. Following assessment by a clinician, most received a diagnosis of “suicide ideation,” and “social problems” was the most commonly used code to record attendance. Following this, patients were typically admitted to an inpatient ward or seen as an outpatient, and then referred to CAMHS. Regarding the potential impact of the COVID-19 pandemic, slightly more females and slightly less males presented at the ED after March 2020. In the year following the pandemic, there were significantly more CYP who were known to a social worker and currently under CAMHS, whereas other services or specialties were used significantly less as a referral pathway. There were also more CYP presenting with self-harm in addition to suicide ideation after the pandemic began.

### Socio-Demographic Characteristics

Our findings are consistent with existing evidence regarding the individual factors associated with suicidality in CYP, including previous self-harm and a history of mental health difficulties (42, 43). Furthermore, the mean age of attendees (13.5 year old) is in line with the notion that early adolescence is a critical period for the onset of mental health conditions, with current statistics suggesting that 50% of lifetime difficulties are first experienced by 14 years of age (44). However, while the higher rate of girls relative to boys presenting in the ED in this study is counter-intuitive based on existing evidence highlighting that men, particularly young men, are more likely to die by suicide than women (45), this may be explained by the gender paradox in suicidal behavior. For example, it is thought that while young men have low rates of suicidal behavior relative to women, they have higher rates of suicide mortality (46); in other words, men are more likely to use violent means and have more serious intentions to die when attempting suicide (47). Thus, they may be less likely to present in the ED looking for help, and their attempts may be more likely to result in death before they can receive support. Women are also more likely to seek help for mental health conditions generally (48, 49) and are more likely to experience internalizing difficulties (e.g., depression/anxiety) commonly associated with suicidality (50, 51), which may also help to explain the gender discrepancy identified here.

An unanticipated and particularly noteworthy finding in this sample is the relatively large proportion [37 vs 1.76% in the wider population; (52)] of CYP who were diagnosed with ASC or suspected to have ASC traits, and specifically those who were autistic girls [61 vs 33% of autistic individuals in the wider population are girls/women; (53)]. There is some evidence to suggest that autistic adults are at greater risk of suicidal ideation and behaviors, including deaths, relative to the rest of the population [0.31 vs 0.04% premature death by suicide; (17, 54–57)]. Indeed, a Swedish mortality study showed a sevenfold increased risk of premature death by suicide in people with ASC compared to the general population (58). However, one study by Hannon and Taylor (59) examining suicidal behavior among autistic young people found that rates were similar in comparison to the general population, with overlapping risk factors. While they initially suggested that ASC traits might be risk factors (e.g., social and communication difficulties may lead to interpersonal problems and social isolation), another study with adolescents and young adults found that it was those with less “severe” autistic traits that were at heightened risk, potentially due to their better emotional insight and more contact with others putting them at increased risk of distress and peer victimization (59, 60).

This hypothesis may lend support for the high presentation rates of autistic girls in the current study, as they are more likely to adopt masking behaviors and engage in social activities than autistic boys (61), which may put them at heightened risk of suicidality. However, one study by Hedley et al. (62) found that while social support and loneliness predicted suicide ideation in autistic individuals, the pattern of relationships in their path analyses was nearly identical for males and females. Thus, this casts doubt on the social interaction hypothesis. Conversely, diagnosis stage may provide an alternative explanation. Girls are more likely to be diagnosed with autism at a later age than boys (19, 63), so there is potential that the delayed diagnosis and subsequent lack of attribution for their experiences may be causing girls heightened levels of distress (60, 64), although more research is first needed before this hypothesis can be confirmed. However, based on the findings identified here, autistic CYP, particularly girls, are a vulnerable group at an increased risk for suicidal crisis, and there is a clear need for greater support and urgency for further research into risk detection and prevention of suicide in autistic people. Increased acceptance of autistic CYP in schools and social groups, and greater awareness and flexibility for autistic CYP, will benefit everyone in society and may also lead to a decrease in feelings of rejection and suicidal thinking for autistic individuals (57). Schools, primary care, and CAMHS services need to be aware of this risk, and should be delivering targeted and effective interventions and services for autistic CYP, in order to prevent them from reaching the point of crisis.

In terms of familial characteristics, high rates of CYP in this sample reported parental or familial difficulties, many of which have previously been identified in the international literature as common risk factors for suicidal ideation or behaviors. For instance, these findings are in keeping with reports from Taliaferro and Muehlenkamp (29) that factors associated with abuse, parental substance misuse, and familial dysfunction or violence are related to adolescent suicide attempts. Furthermore, Perkins and Hartless (27) found a clear association between abuse and frequent suicidal thoughts and attempts for all adolescents, regardless of gender or ethnicity, although factors such as parental substance abuse and family structure were not significant once other risk factors were accounted for. However, another study of adolescents hospitalized for suicidal ideation and/or behavior did find that poorer perceptions of family functioning by adolescents were positively associated with suicide ideation and history of suicide attempts (65). Similarly, a study by Thompson et al. (66) in the United States, found that the presence of familial adverse childhood experiences (ACEs), including abuse, parental incarceration, and family history of suicidality in childhood increased the odds of later suicide attempts or ideation, with the accumulation of multiple ACEs further increasing the odds. Thus, while our findings are in keeping with previous international literature, this is one of the first times that familial factors associated with suicidal crisis in a clinical sample of CYP have been examined in a United Kingdom setting. The characteristics identified here can be used to help inform early identification and intervention with CYP who may be at heightened risk of experiencing suicidal crisis, although further studies with larger samples are still needed.

### Coding Practices

Eight different codes were used by clinicians when recording patients who presented in the ED with suicidal crisis, the most common of which was “social problems.” Perhaps unsurprisingly, codes of “overdose” and “deliberate self-harm” were used more frequently for CYP who had presented with suicidal ideation and self-harm. However, few other significant predictors of code use were identified. The heterogeneity in coding practices found here is a cause for concern that has been flagged in several other reports recently, as current data may be misrepresentative of the true volume of suicidal crisis presentations [e.g., (33, 67)]. Given that EDs are often the first point of contact for people experiencing distress relating to suicidality (68), it is important that we have a system that can provide an accurate indication of the number and characteristics of individuals who are presenting in crisis, in order to be able to offer tailored and effective levels of support.

A “suicidal thoughts” code is available for use (R45.81) in the current coding system (International Classification of Diseases 10^th^ Edition; ICD-10); however, in the present sample, it was only used 10% of the time, despite all patients in the study having attended ED with “suicide ideation” as their primary diagnosis. It appears that this is not a unique issue, with other international studies reporting similar findings. For instance, research conducted by Sveticic et al. (67) in Australia also found great variation in code use for suicide ideation, with 38 different codes used, and the R45.81 code being used less than half the time. Another study by Anderson et al. (69) in America reported the same issue, with only 3% of patients with an indication of suicidal ideation in the notes field having a corresponding ICD-9 code. While the exact reason for the coding issue in the current study is unknown, it may be due to the ICD-10 guidelines stating that this code should only be used if the clinician is certain there is no underlying mental disorder, meaning that other codes are chosen. Further research is needed in this area, to determine clinicians’ reasoning for code choices, and there is a clear need for improved coding practices relating to suicidal crisis (33).

### Limitations

This study has several strengths and provides significant contributions to the extant evidence base, by helping to paint a picture of the CYP who are seeking help from EDs for suicidal crisis in North-West England. This information can help to inform early identification strategies to determine those who may be at-risk, meaning effective intervention and support strategies can be offered, and subsequent distress prevented. However, these findings should be interpreted within the context of some methodological limitations. Firstly, the relatively small sample size limits the statistical power, and effect sizes are also generally small, meaning that any significant findings should be interpreted with caution, and analytic techniques are limited in their complexity. The sample is also limited to CYP who are seeking help, and thus likely only reflects a small minority of CYP who are experiencing suicidal crisis. Furthermore, as a case series study, there was no control group, meaning that true risk factors cannot be identified. Comparative data would highlight the socio-demographic characteristics that puts some CYP at heightened risk of suicidal crisis, relative to the rest of the population. In addition, data were only collected from a single hospital in one region of England over a 2-year period. Thus, care must be taken when attempting to generalize these findings to other geographical regions. It is also important to note that the variables available for analysis were limited to those self-reported by attendees, their parents, and/or clinicians. Finally, due to its retrospective nature, some patient data have been inevitably missing.

Notwithstanding these limitations, this was a highly exploratory study, aiming to gain insight into and understanding of patient profiles and pathway management. Thus, this provides a basis for further work in this area, utilizing larger samples, comparison groups, and multiple NHS Trusts across various regions of the United Kingdom.

### Conclusion

Our results provide the first clear indication in the United Kingdom of the common socio-demographic characteristics of CYP in North-West England who present in EDs seeking help for suicidal crisis, and the resultant care pathways that they follow. The findings highlight the high proportion of attendees who were White and female, and who were diagnosed with or suspected to have autism, as well as inconsistencies in the coding practices used when recording patient attendances. Thus, our findings have implications for early identification and intervention with children who may be at a heightened risk for suicidal crisis, and for the development of guidance surrounding the coding practices that need to be used. In turn, this will help to ensure an accurate understanding of the rates of suicidal crisis among CYP in the United Kingdom, and will support the development of effective services for CYP. However, we acknowledge the highly explorative nature of this study, and call for similar research to be conducted on a larger scale in order to confirm our findings.

## Data Availability Statement

The data analyzed in this study is subject to the following licenses/restrictions: Data are sensitive and confidential. Requests to access these datasets should be directed to EA, e.l.ashworth@ljmu.ac.uk.

## Ethics Statement

Ethical review and approval was not required for the study on human participants in accordance with the local legislation and institutional requirements. Written informed consent for participation was not required for this study in accordance with the national legislation and the institutional requirements.

## Author Contributions

EA led the project, analyzed the data, and wrote the manuscript. SP collected and analyzed the data. MM analyzed the data and supported the writing of the manuscript. PS supported with the interpretation of the findings and the writing of the manuscript. All authors read and provided feedback on manuscript drafts.

## Conflict of Interest

The authors declare that the research was conducted in the absence of any commercial or financial relationships that could be construed as a potential conflict of interest.

## Publisher’s Note

All claims expressed in this article are solely those of the authors and do not necessarily represent those of their affiliated organizations, or those of the publisher, the editors and the reviewers. Any product that may be evaluated in this article, or claim that may be made by its manufacturer, is not guaranteed or endorsed by the publisher.
